# Unexpected Histopathological Diagnosis of Undifferentiated Uterine Sarcoma after Simple Hysterectomy: Extrapolating Limited Evidence

**DOI:** 10.7759/cureus.6783

**Published:** 2020-01-27

**Authors:** Amenda Ann Davis

**Affiliations:** 1 Obstetrics and Gynecology, All India Institute of Medical Sciences, New Delhi, IND

**Keywords:** sarcoma, uterine sarcoma, undifferentiated uterine sarcoma, leiomyosarcoma, uterine neoplasms/therapy, combined modality therapy, leiomyosarcoma/therapy, gynecologic surgical procedures

## Abstract

Uterine sarcomas are a rare malignancy, often retrospectively diagnosed after myomectomy or hysterectomy. Undifferentiated uterine sarcomas (UUS) are a particularly aggressive variant of this condition. Little evidence exists regarding the postoperative management of undifferentiated sarcomas diagnosed after hysterectomy performed for presumed benign conditions. We describe the case of a 33-year-old woman who presented with heavy bleeding and subsequently underwent hysterectomy on an emergency basis after failed medical management. Cut-section of the uterus revealed a grossly benign-looking sub-mucosal fibroid. However, the final histopathology report revealed undifferentiated uterine sarcoma. We worked up the patient postoperatively with MRI to rule out metastasis and performed bilateral salpingo-oophorectomy based on hormone receptivity status. We followed this with single-agent chemotherapy with adriamycin, which was followed by continuous therapy with oral letrozole (aromatase inhibitor). The patient was found doing well at the two-year follow-up, with no evidence of relapse. Postoperative diagnosis of UUS should include imaging to rule out metastasis, consideration for completion of surgery based on hormone receptivity of tumour, and lymphadenectomy based on the subtype of tumour.

## Introduction

Uterine sarcomas are a rare malignancy and often a post-operative diagnosis. High-grade undifferentiated uterine sarcomas (UUS) are an aggressive subtype of uterine sarcomas and are often associated with poor prognosis [1]. The preoperative diagnosis of uterine sarcoma is difficult, owing to the low sensitivity of tests such as imaging, biopsy, and cancer markers. It requires a high degree of suspicion and is often an unexpected postoperative histopathological diagnosis. We describe the case of a 33-year-old woman who presented with acutely heavy bleeding, which precluded a complete preoperative workup. We performed emergency hysterectomy due to non-responsive acute menorrhagia. Postoperatively, she was diagnosed with undifferentiated uterine sarcoma (UUS), with positivity for estrogen receptor (ER) and progesterone receptor (PR). We describe the successful postoperative management of this case by the application of limited available evidence for this type of malignancy.

## Case presentation

Our patient was a 33-year-old homemaker who presented in the emergency with the chief complaint of heavy vaginal bleeding for the past 14 days. Since her menarche at the age of 11 years, she had had regular menstrual periods with average flow. However, for the past one year, she had been suffering from heavy, prolonged menses, lasting 15-20 days and producing large clots. She also complained of the descent of a vaginal mass, which she felt was more evident during intercourse and attributed it to the post-coital bleeding she had been experiencing for the past few months. There was no increase in the size of the mass after prolonged standing, straining during defecation, or micturition. She had no history of abdominal pain, abdominal mass, urinary or bowel complaints, easy bruising, excess fatigue, or weight loss. She had been advised tranexamic acid orally and had been taking 3 g per day. She had been married at the age of 18 years, had had four term vaginal deliveries, and had never used any form of contraception in the past. For the past 14 days, her bleeding had been more profuse than usual, with episodes of flooding; and tranexamic acid had provided minimal or no relief. She had been then referred to us, a tertiary centre, for subsequent management.

We found her to be extremely pale, with a pulse rate of 102/minute, and a blood pressure of 92/64 mm. General physical examination was otherwise insignificant. Her abdomen was soft with no organomegaly. On per speculum examination, the vagina was found to be entirely filled with a large, irregular 8x8-cm mass coming out of the cervix. It bled on touch, but there were no areas of necrosis or ulceration. There was no foul odour. On per vaginal examination, the mass was firm, occupying the entire vagina, and non-tender. Her uterus could not be felt separately, and fornices could not be reached. Her diagnostic workup showed severe anemia and fibroid uterus (Table 1).

**Table 1:** Preoperative investigations

Parameter	Value
Hemoglobin	5.8 gm/dL
Total leukocyte count	8,400/dL
Platelet count	260,000/dL
Ultrasound	Uterus bulky with a well defined 5.2x4.1-cm mass of mixed echogenicity arising from posterior wall of myometrium, pushing central endometrium anteriorly. No evidence of necrosis. Suggestive of fibroid

**Figure 1:**
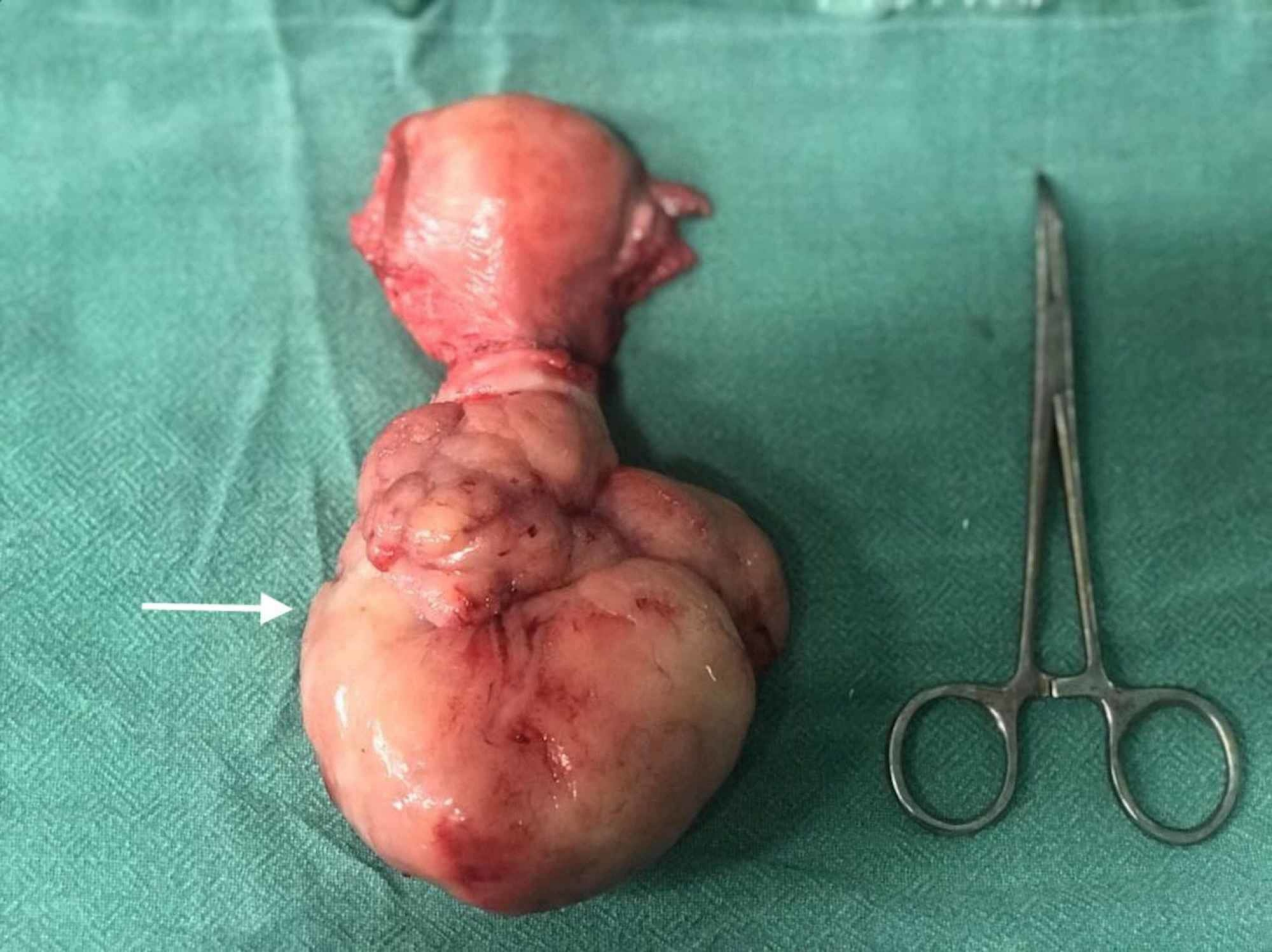
Intra-operative findings during hysterectomy Uterus was bulky with an 8x8-cm fibroid protruding from the cervix (white arrow)

**Figure 2:**
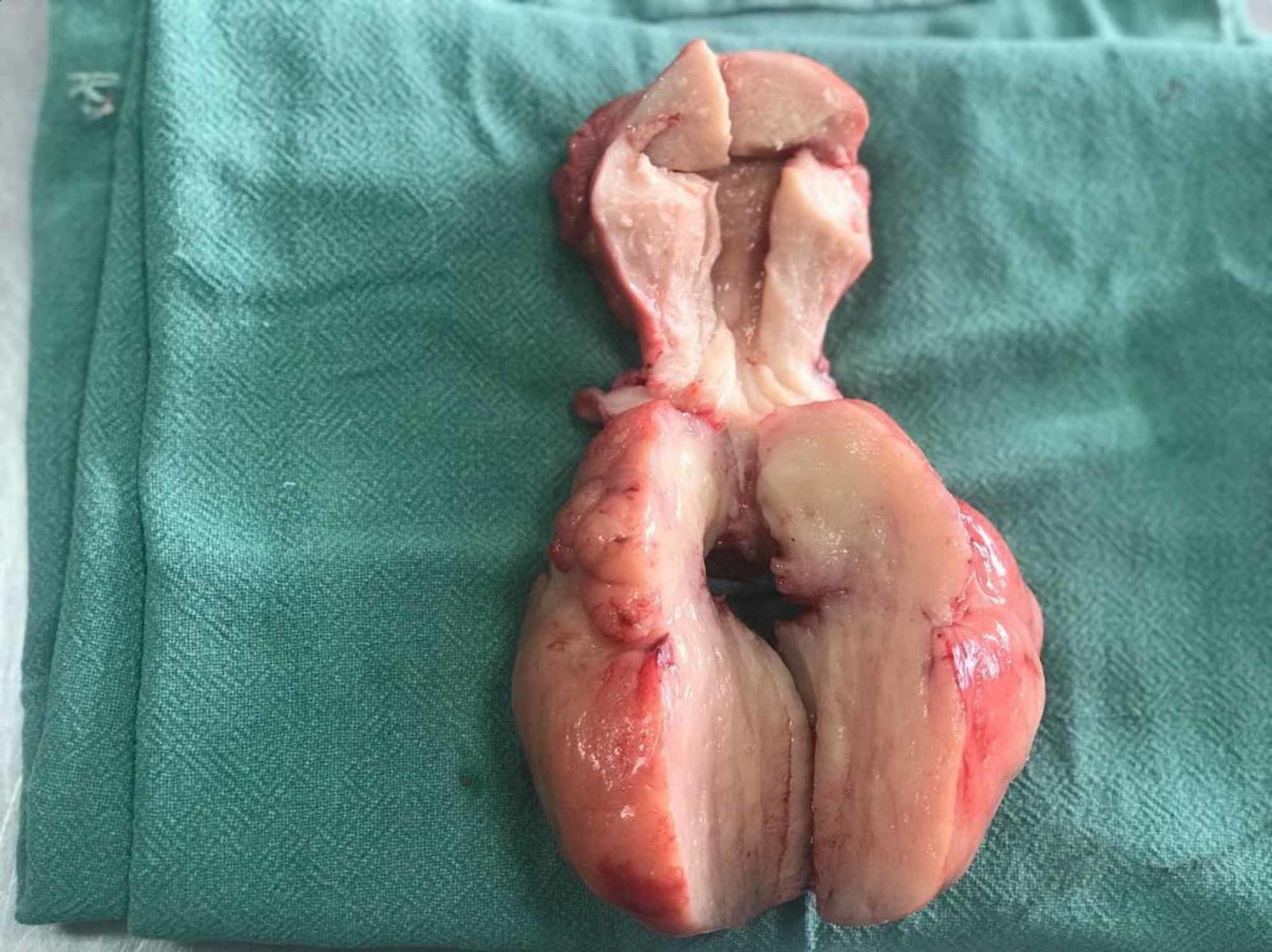
Cut-section of the uterus

Our primary diagnosis was a sub-mucosal (type 0 or 1 fibroid according to the International Federation of Gynecology and Obstetrics classification) [2]. We attempted to control the bleeding with intravenous tranexamic acid and a high dose of oral norethisterone, but our patient continued to have torrential bleeding. We took her for an emergency total abdominal hysterectomy on the same day. Intra-operatively, the corpus of the uterus was bulky, with an 8x8-cm fleshy lobulated fibroid polyp protruding from the cervix (Figure 1), which was smooth and regular on cut-section (Figure 2). The patient received three units of packed red blood cells postoperatively and was discharged after two days.

On histopathological examination, a gross evaluation showed a total hysterectomy specimen with a 9x6-cm polypoidal growth from the posterior isthmus, extending into the cervix, which was fleshy, white, and congested, but with no necrosis or haemorrhage. The uterine fundus was free of tumour. Microscopy showed spindle-shaped cells with eosinophilic cytoplasm, prominent nucleoli, marked nuclear pleomorphism, brisk mitosis, and giant tumour cells. Immunohistochemistry was positive for CD10, cyclin D1, desmin, ER and PR and negative for pan-cytokeratin, epithelial membrane antigen, smooth muscle actin, and myogenin. The expert pathological review was confirmatory of high-grade UUS, stage IB. 

To guide further management, we obtained a pelvic and abdominal MRI with gadolinium contrast, which showed no evidence of residual tumour or lymph-node involvement. We performed laparoscopic bilateral salpingo-oophorectomy three weeks after the first surgery, in light of ER and PR positivity. We then administered three cycles of single-agent chemotherapy with adriamycin (doxorubicin) at the dose of 75 mg/m^2^ IV, followed by continuous therapy with the aromatase inhibitor letrozole 2.5 mg/day. At the two-year follow-up, the patient was found doing well, with no evidence of relapse.

## Discussion

Uterine sarcomas are rare mesenchymal tumours accounting for 3% of endometrial cancers. The most common are leiomyosarcomas (LMS), which account for 60%, followed by endometrial stromal sarcomas (ESS; 20%). Our patient had an undifferentiated or poorly differentiated uterine sarcoma, the least common of uterine sarcomas [1]. Sarcomas can have a variety of clinical presentations, most commonly post-menopausal bleeding or pre-menopausal abnormal uterine bleeding, which was our patient’s primary symptom. Uterine sarcoma is often a retrospective, histological diagnosis obtained after resection of the uterus by myomectomy or hysterectomy. Since sarcomas are relatively rare, it is difficult to pinpoint the risk factors. They are usually seen in older age groups but can occur in women as young as 20 years of age [3]. The United States Food and Drugs Administration (USFDA) has issued a black-box warning stating that tamoxifen is a definite risk factor for developing LMS [4].

One of the questions pertinent to this case is whether we could have diagnosed or suspected uterine sarcoma preoperatively. This is often a difficult task, even with a prudent combination of history and examination, imaging, biopsy, and tumour markers. In a retrospective analysis of 1,332 women undergoing hysterectomy for fibroid, 0.27% of the 371 patients who had a history of rapid growth had a final diagnosis of sarcoma; whereas 0.15% of 961 who did not have any such history was diagnosed with sarcoma. This refuted an earlier theory that rapid uterine growth (6-weeks' size) within one year suggested sarcoma [5]. A more recent meta-analysis of 26 studies with 580 women showed that a history of rapid growth was documented in only 2.6% [6]. The only exception may be rapidly growing or extremely large fibroids in postmenopausal women, which must be dealt with more cautiously. Imaging modalities that have been found to be useful in the diagnosis of uterine sarcomas are ultrasound, positron emission tomography (PET), and MRI, but not CT [7]. On sonography, we may see mixed echogenic and poor echogenic parts, central necrosis, and irregular vessel distribution, low impedance to flow, and high peak systolic velocity on doppler, but these findings may prevail in benign fibroids as well [8]. MRI may show intralesional haemorrhage, necrosis, ill-defined margins, and consistent absence of calcifications. High-signal intensity has not been found to be reliable [8]. In a study by Goto et al., preoperative MRI with gadolinium diethylenetriaminepentaacetic acid (Gd-DTPA) contrast‐enhanced dynamic MRI had a diagnostic accuracy of 0.971, with a sensitivity of 1.0, and specificity of 0.969 in 227 cases of uterine tumours, 10 of which were LMS [9]. Sato et al. suggested an algorithm using a combination of diffusion weight imaging and apparent diffusion coefficient, stratifying patients into low- and high-risk groups for LMS with 100% sensitivity, 94.0% specificity, and 66.7% positive predictive value [10]. PET scan may reveal increased 18-fluorodeoxyglucose uptake, but this is reduced in low-grade tumours. Moreover, benign tumours can also have increased uptake [8]. Endometrial biopsy has been shown to be diagnostic in 33-68% of sarcomas (low sensitivity), with no difference irrespective of whether endometrial aspiration or dilatation and curettage is performed [11]. Tumour markers such as Ca-125 and lactate dehydrogenase (LDH) isozyme 3 may be elevated in sarcomas, but the quality of such evidence is very low [12]. Nagai et al. developed PREoperative Sarcoma Score (PRESS) incorporating endometrial cytology/biopsy, intra tumour high-intensity signals on T2 MRI, serum LDH, and age, to predict preoperative sarcoma with 84.1% accuracy [13]. All of the above diagnostic modalities require a degree of suspicion, as we routinely do not apply multiple diagnostic modalities to women with fibroids, particularly to young women like in our case.

The subsequent dilemmas which arise post-diagnosis include uncertainty about performing salpingo-oophorectomy, lymphadenectomy, radiotherapy, and other adjuvant therapy. There is extremely limited literature specific to UUS; hence we extrapolated findings from other types of uterine sarcomas. Adjuvant radiation therapy has been retrospectively found to be associated with poor survival in uterine sarcomas, leading us to rule it out it for our patient [14]. Regarding ESS, ovarian ablation is needed in estrogen-positive tumours as estrogen acts as a trophic agent, with better outcomes after bilateral versus unilateral and no salpingo-oophorectomy [15,16]. As our patient was ER-positive, we opted for bilateral salpingo-oophorectomy. UUS is reportedly a more aggressive tumour than ESS and LMS, with a higher incidence of lymph node metastasis [17]; however, due to lack of evidence and radiologically normal nodes, we did not perform lymphadenectomy for our patient. Undifferentiated sarcomas usually do not express hormonal receptors, while LMS shows estrogen receptivity in 18-87% and EES in 40-100% [18]. Hence, we modified the recommendations of the National Comprehensive Cancer Network (NCCN), by extending the recommendation of hormone suppression therapy to UUS and putting our patient on letrozole [19]. Because of this hormone-receptor positivity, hormone replacement therapy was contraindicated, and strong counseling was required about menopausal symptoms and sexual health, including the use of lubricants and moisturisers. 

The evidence on the management of UUS is very limited and is often based on the recommendations on other types of sarcomas with shared characteristics. Uterine sarcomas can occur at any age and require a very high degree of suspicion and multiple diagnostic modalities to predict preoperatively. MRI is considered the most useful modality currently. Bilateral salpingo-oophorectomy should be done if the ER is positive. Hormone suppression therapy in the form of aromatase inhibitors should be strongly considered for ER-positive sarcomas, irrespective of the subtype. There is inadequate evidence for or against lymphadenectomy for UUS.

## Conclusions

High-grade UUS is a rare and often unexpected postoperative diagnosis. This case demonstrates how careful application and extrapolation of evidence can lead to successful patient outcomes.
